# Classification of *Proteus penneri* lipopolysaccharides into core region serotypes

**DOI:** 10.1007/s00430-016-0468-8

**Published:** 2016-07-28

**Authors:** Agata Palusiak

**Affiliations:** Department of General Microbiology, Institute of Microbiology, Biotechnology and Immunology, University of Łódź, Banacha 12/16, 90-237 Lodz, Poland

**Keywords:** Classification scheme, Core region, Core serotype, Lipopolysaccharide, *Proteus penneri*

## Abstract

The frequency of *P. penneri* isolation from hospital patients, mostly from urine and wounds, keeps on growing, and numerous isolates are multi-drug resistant. *P. penneri* rods produce lipopolysaccharide (LPS), which may lead to the septic shock. Until now, O-specific polysaccharide has been the best structurally and serologically characterized region of *P. penneri* LPS. It is worth having an insight into the serological specificity of both poly- and oligosaccharide parts of *P. penneri* LPS. The *P. penneri* core region is less structurally diverse than OPS, but still, among other enterobacterial LPS core regions, it is characterized by structural variability. In the present study, the serological reactivity of 25 *P. penneri* LPS core regions was analyzed by ELISA, passive immunohemolysis and Western blot technique using five polyclonal *P. penneri* antisera after or without their adsorption with the respective LPSs. The results allowed the assignment of the tested strains to five new core serotypes, which together with published serological studies led to the creation of the first serotyping scheme based on LPS core reactivities of 35 *P. penneri* and three *P. mirabilis* strains. Together with the O types scheme, it will facilitate assigning *Proteus* LPSs of clinical isolates into appropriate O and R serotypes.

## Introduction

*Proteus penneri*, previously designated as *P. vulgaris* biogroup 1, was identified and named in 1982 by Hickman et al. [1] on the basis of low DNA relatedness to DNA of the biogroups 2 and 3 representatives and its phenotypic differences. Although these Gram-negative, peritrichously flagellated rods are less common among *Proteus* spp. clinical isolates than *P. mirabilis* strains (70–90 % of *Proteus* spp. infections) [2, 3], the frequency of their isolation from hospital patients keeps on growing [2, 4] and misidentification may further contribute to a lowered number of *P. penneri* isolation reports [3, 5]. The most common body sites of *P. penneri* strains isolation are wounds (of abdomen, foot, groin, hip and neck) and the urinary tract, especially of long-term catheterized patients or individuals with anatomical abnormalities within the tract [2, 4, 6, 7]. *P. penneri* strains were also isolated from: blood, fecal specimens, ankle ulcer, sacral decubitus, conjunctiva, subcutaneous thigh or cerebral abscess, skin lesion aspirate, abdominal drain fluid, diabetic foot ulcer, bronchoalveolar lavage fluid, a pulmonary artery catheter tip, cerebrospinal fluid, sputum and the center of struvite bladder stone [2–4, 6, 7].

**Fig. 1:**
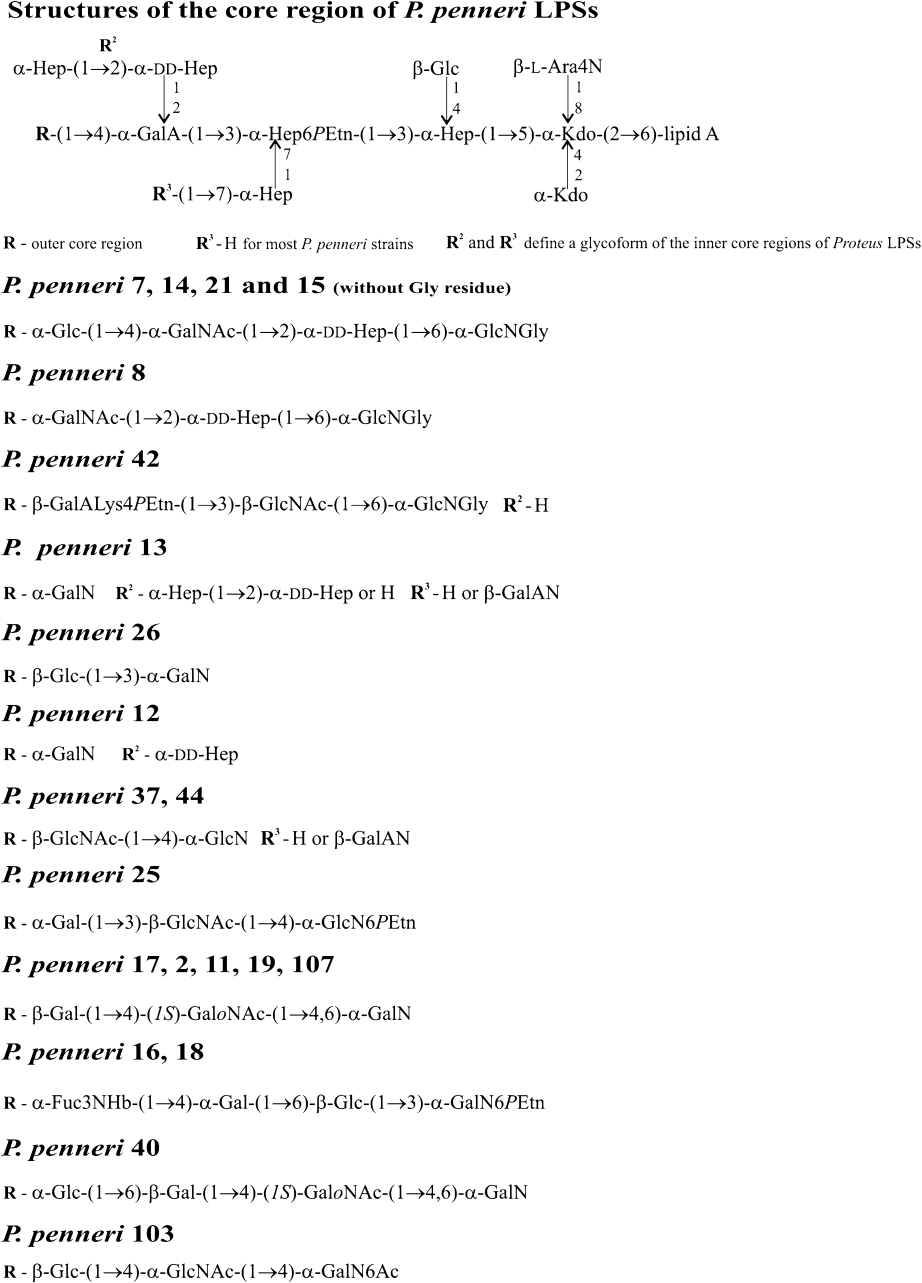
Structural variability of *P. penneri* LPS core regions [11]; Ara*p*4N, 4-amino-4-deoxy-l-arabinopyranose; Fuc3NHb, 3-[(*R*)-3-hydroxybutyryl]amino-3,6-dideoxy-d-galactose, Gal, galactose; GalA, galacturonic acid; GalALys, amide of GalA with lysine; GalAN, amide of GalA with aliphatic polyamines; GalN, galactosamine; GalNAc, 2-acetamido-2-deoxy-d-galactose; (*1S*)-Gal*o*NAc, open-chain form of GalNAc; GalN6Ac, 6-*O*-acetyl-2-amino-2-deoxy-d-galactose; Glc, glucose; GlcNAc, 2-acetamido-2-deoxy-d-glucose; GlcNGly, 2-glycylamino-2-deoxy-d-glucose; Hep, l-*glycero*-d-*manno*-heptose; dd-Hep, d-*glycero*-d-*manno*-heptose; Kdo, 3-deoxy-d-*manno*-oct-2-ulosonic acid; and *P*Etn, 2-aminoethyl phosphate

*P. penneri* produce many virulence factors which enable them to cause infections, e.g., urease, fimbriae and hemagglutinins, hemolysins, metalloproteases, flagella, siderophores and lipopolysaccharide (LPS) [2, 4]. LPS consists of three structurally different regions: lipid A (defined structurally only for one *P. mirabilis* mutant), core oligosaccharide (OS) and O-specific polysaccharide (OPS) [4, 8]. Until now, OPS has been the best structurally and serologically characterized region of *P. penneri* LPS, which also defines the serospecificity of smooth bacterial cells. Twenty-six different OPS structures have been identified for *P. penneri* strains so far, among which seven are common also to the other representatives of the genus [4, 9, 10]. The *P. penneri* core region is less structurally diverse than OPS but in contrast to other enterobacterial LPS core regions characterized by lager structural variability. Up to date, 12 different structures of the outer core region, accounting for the structural diversity of the *P. penneri* LPS core regions, were identified (Fig. 1) [4, 11]. The majority of tested *P. penneri* strains presented one major glycoform of the inner core region [11, 12] (Fig. 1). There are only two strains, *P. penneri* 12 and 42, which present glycoforms of the inner core region not identified in any other *Proteus* spp. LPSs [4, 11, 12]. Moreover, the heterogeneity of this LPS part may appear also within one strain, e.g., *P. penneri* 13 forms ten variants of its core-lipid A backbone [4]. The *P. penneri* classification scheme is based on the OPSs serospecificity. So far, *P. penneri* isolates have been classified into 17 *Proteus* O serogroups, among which 13 consist of these species representatives only [4, 9, 10, 13]. To have an insight into the serological specificity of both polysaccharide and oligosaccharide parts of *P. penneri* LPS, it is worth creating an additional scheme classifying *P. penneri* LPSs into serotypes of their core regions. A core types classification scheme which together with the O-types scheme may serve as a diagnostic tool facilitating the assignment of *Proteus* LPSs of clinical isolates into appropriate O and R serotypes. In the current work, the results of serological studies prove the existence of another five serotypes of *P. penneri* core regions, which is evidence of further structural variations within this part of *Proteus* spp. LPS.

## Materials and methods

### Bacterial strains and LPSs

*P. penneri* 2 (O66), 11, 12 (O58), 16, 18 (O17), 17 (O8), 19, 24 (O64a,b,c), 28 (O31a,b), 31 (O19a,b), 35, 36 and 38 (O64a,b,c) were kindly provided by Prof. D. J. Brenner, Center for Disease Control and Prevention in Atlanta (USA); *P. penneri* 100 (O64a,b,c), *P. penneri* 103 (O73a,b), 107 (O8), 114 (O64a,b,c), 115 (O58) and 124 (R form) were from Dr. B. Holmes (National Collection of Type Cultures, London, UK); and *P. penneri* 60 (O70), 63 (O68) and 75 (O73a,c) were isolated from the urine of patients with bacteriuria in a Łódź hospital. All strains are stored in glycerol at −80 °C at the Department of General Microbiology, University of Łódź.

The *P. penneri* 18 LPS was isolated by the phenol-water procedure according to the Westphal and Jann method (1965) and purified with aqueous 50 % trichloroacetic acid [14].

*P. penneri* 2, 11, 12, 16, 17, 19, 24, 26, 28, 31, 35, 36, 38, 60, 63, 75, 100, 103, 107, 112, 114, 115 and *P. vulgaris* 55/57 LPSs have been previously obtained by the Westphal and Jann method [14], and *P. penneri* 13 and 124 LPSs, by the phenol/chloroform/petroleum ether method of Galanos (1969) [15]. These LPSs were from the collection of the Department of General Microbiology.

Alkali-treated LPSs used for passive immunohemolysis (PIH) were obtained by LPS saponification with 0.25 M NaOH (2 h, 56 °C) and precipitation with 96 % ethanol [16].

### The sera

Rabbit polyclonal sera against the whole cells of *P. penneri* 17, 28, 60, 103 and 124 came from the collection of the Department of General Microbiology.

### Serological assays

LPSs samples were checked with the appropriate antiserum in the enzyme-linked immunosorbent assay (ELISA), in Western blot after sodium dodecyl sulfate-polyacrylamide gel electrophoresis (SDS-PAGE) and/or in PIH according to the previously described procedures [17, 18]. For PIH, sheep red blood cells (SRBCs) were sensitized with alkali-treated LPSs (64–200 μg/0.2 ml of SRBCs); 50 ng of LPS per well was used for coating microtiter plates in ELISA. The highest dilution of antiserum giving optical density 405 = 0.2 was assumed as the antibodies titer.

### Adsorption procedures

- By alkali-treated LPSs

Single antiserum diluted 1:50 with a veronal buffer (pH 7.3) was incubated for 30 min on ice with SRBCs (0.2 ml) sensitized with 200 μg of appropriate alkali-treated LPS from one *Proteus* spp. strain. The antiserum titer was determined by PIH as the last antiserum dilution resulting in 50 % hemolysis [16].

- By the LPS on bacterial cells

This adsorption procedure was performed in the case of i.a. the *P. penneri* 17 antiserum due to the weak activity of cross-reacting alkali-treated LPSs in PIH compared to activity of the native LPSs in ELISA.

A wet mass of bacterial cells, after being washed in phosphate-buffered saline (PBS), was suspended in serum diluted 1:100 in PBS, incubated for 30 min on ice and removed from the serum by centrifugation.

Each serum was adsorbed three–four times to make sure that all antibodies that were able to bind to LPS molecules were removed from the serum.

## Results

In previous serological studies, sera specific to appropriate *P. penneri* strains were tested with a set of 40 *P. penneri* LPSs and the core region serospecificity for the majority of those antigens was determined [16, 17, 19, 20]. In the present work, the reactivities of the core regions of *P. penneri* LPSs 2, 11, 12, 13, 16, 17, 18, 19, 24, 26, 28, 31, 35, 36, 38, 60, 63, 75, 100, 103, 107, 112, 114, 115 and 124 with five sera specific to the strains *P. penneri* 17, 103 (with defined structures of LPS core regions [11]), 28, 60 and 124 were analyzed by ELISA and Western blot and classified into core serotypes. Each serum was adsorbed with a single cross-reacting antigen and tested again in PIH or in ELISA (*P. penneri* 17 antiserum) with all LPSs reacting with the serum to exclude further serological differences within the groups.

### *P. penneri* 28 antiserum

**Fig. 2:**
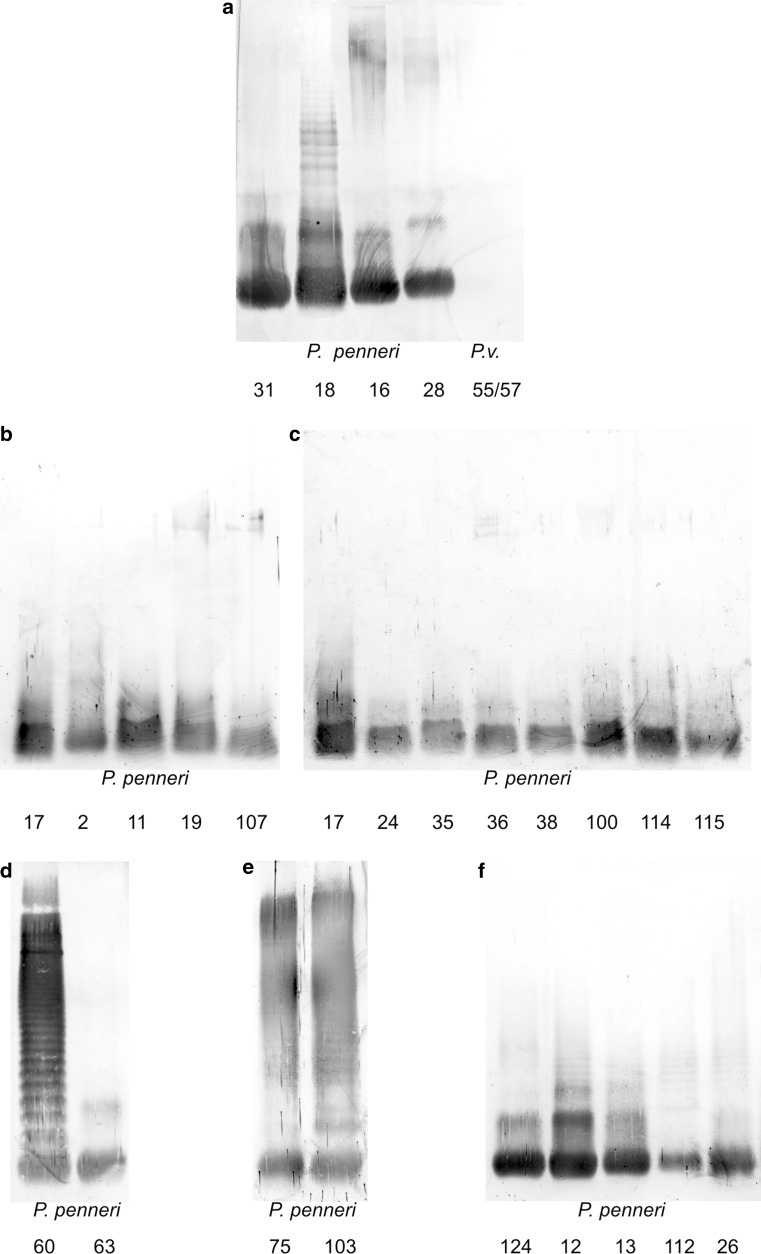
Western blot data of *Proteus* spp. LPSs with antisera against: **a** *P. penneri* 28 (anti-core serum), **b**, **c** *P. penneri* 17, **d** *P. penneri* 60, **e** *P. penneri* 103 and **f** *P. penneri* 124

*P. penneri* 28 antiserum has been obtained by immunizing with whole bacterial cells. O-polysaccharide-specific immunoglobulins were eliminated from the serum by its adsorption with an alkali-treated LPS of *P. vulgaris* 55/57 (O31a,b) containing the OPS structurally identical to *P. penneri* 28 OPS and a different core region serotype [21]. A lack of reaction with *P. vulgaris* 55/57 LPS indicated that all antibodies specific to the OPS were completely removed from the serum (Fig. 2a). This antiserum will be referred to as anti-core serum. Three LPSs, *P. penneri* 16, 18 and 31, reacted with this serum in ELISA of which *P. penneri* 31 was distinguished by the lowest reactivity titer (Table 1). In Western blot, *P. penneri* 28 anti-core serum bound to low-molecular-mass LPS species (consisting of the core-lipid A moieties) of all the LPSs used, among which *P. penneri* 18 LPS showed the strongest and the most distinguishing reactions (Fig. 2a). The ladder-like banding pattern, which can be noticed in Fig. 2a, also corresponds to the low-molecular-mass LPS species of *P. penneri* 18 LPS. This observation was confirmed by the Western blot results obtained for *P. penneri* 18 LPS and antiserum against the *P. penneri* 28 strain after its adsorption with *P. penneri* 18 LPS molecules. The reactions previously observed in Western blot with unadsorbed anti-core serum (Fig. 2a), were abolished. Moreover, *P. penneri* 28 anti-core serum adsorbed with *P. penneri* 16, 18 and 31 LPSs did not react in PIH with any of the antigens used (data not showed).

**Table 1:** Reactivity of *P. penneri* 28 anti-core serum and antisera against *P. penneri* 17, 60, 103 and 124 with the *P. penneri* LPSs in ELISA^a^

LPSs from *P. penneri* strains	Reciprocal titer for LPS in ELISA
*P. penneri* 28 anti-core serum	
*P. penneri* 28	*128.000*
*P. penneri* 16	64.000
*P. penneri* 18	128.000
*P. penneri* 31	32.000
*P. penneri* 17 antiserum	
*P. penneri* 17	*64.000*
*P. penneri* 2	16.000
*P. penneri* 11	64.000
*P. penneri* 19	32.000
*P. penneri* 107	16.000
*P. penneri* 24	16.000
*P. penneri* 35	32.000
*P. penneri* 36	16.000
*P. penneri* 38	16.000
*P. penneri* 100	16.000
*P. penneri* 114	16.000
*P. penneri* 115	8.000
*P. penneri* 60 antiserum	
*P. penneri* 60	*512.000*
*P. penneri* 63	4.000
*P. penneri* 103 antiserum	
*P. penneri* 103	*1024.000*
*P. penneri* 75	1024.000
*P. penneri* 124 antiserum	
*P. penneri* 124	*32.000*
*P. penneri* 12	32.000
*P. penneri* 13	32.000
*P. penneri* 112	32.000
*P. penneri* 26	8.000

^a^Data for the homologous LPSs are italicized

### *P. penneri* 17 antiserum

Although *P. penneri* 17 antiserum is specific to the whole bacterial strains, *P. penneri* 17 LPS did not show in Western blot (Fig. 2b,c) the reaction typical for the high-molecular-mass LPS species containing O-polysaccharide, which suggests that core-specific antibodies dominate in the serum. In ELISA, the *P. penneri* 17 antiserum strongly cross-reacted with *P. penneri* 2, 11, 19 and 107 LPSs (Table 1), which is in accordance with structural studies which showed that all LPSs present one structural type of the core region (Fig. 1) [11]. Further, six LPSs from *P. penneri* 24, 35, 36, 38, 100, and 114 exhibited in ELISA strong cross-reactions, whereas *P. penneri* 115 LPS showed only weak reactivity (Table 1). In Western blot, the reactions of *P. penneri* 17 antiserum, concerning the core-lipid A molecules of all used *P. penneri* LPSs, were similar to the reaction of homologous LPS (Fig. 2b, c). The adsorption of the *P. penneri* 17 antiserum by each tested antigen completely abolished its reactivity with all the LPSs used (data not showed).

### *P. penneri* 60 antiserum

**Fig. 3:**
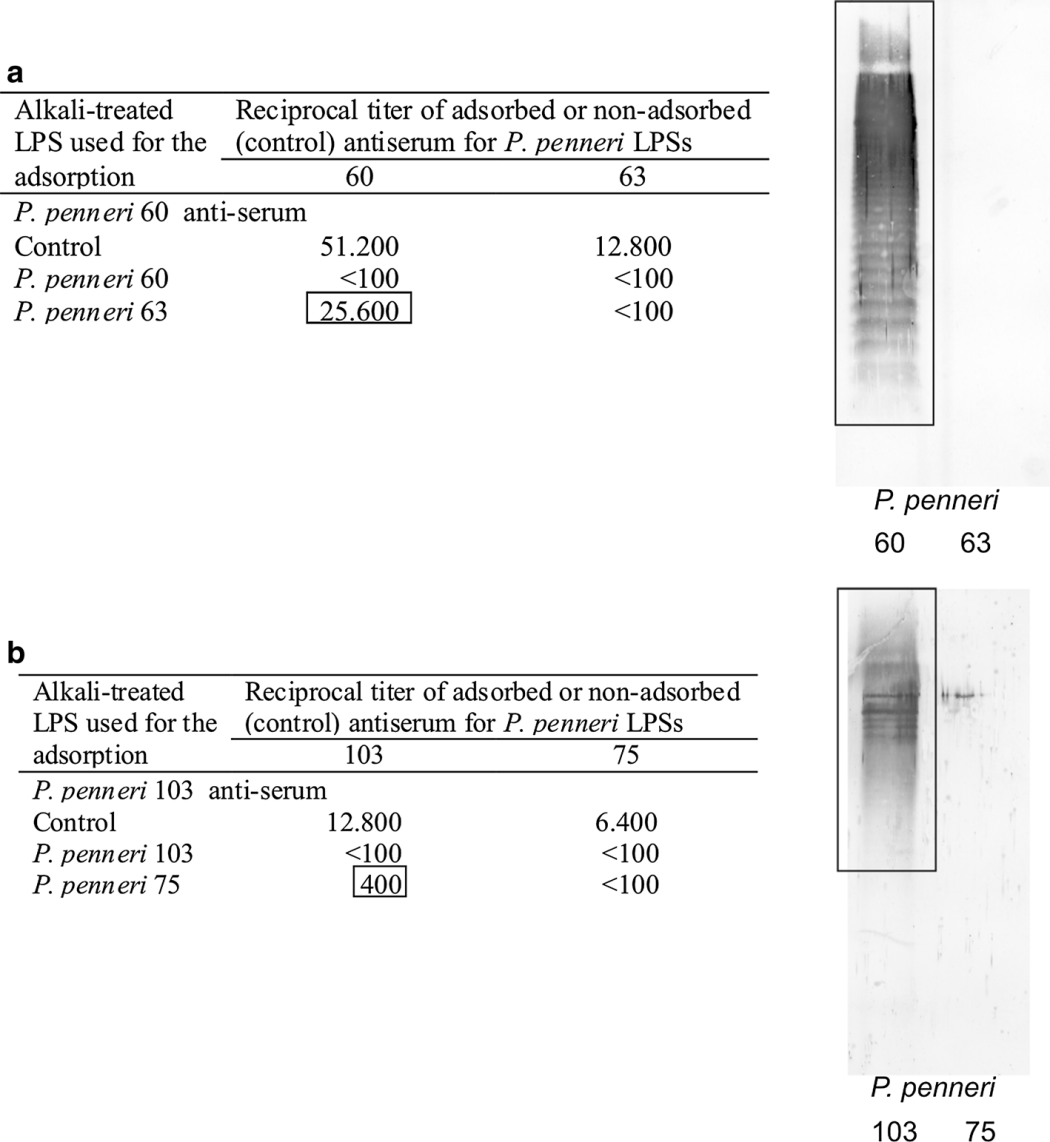
Passive immunohemolysis and Western blot data of the *P. penneri* LPSs with adsorbed antisera against strains: **a** *P. penneri* 60, **b** *P. penneri* 103. The data in frames show the reactions of antibodies remaining in the tested serum after its adsorption by: **a** *P. penneri* 63 LPS molecules, **b** *P. penneri* 75 LPS molecules

In ELISA, anti-*P. penneri* 60 serum cross-reacted with one LPS, *P. penneri* 63, but more weakly than homologous LPS (Table 1). In Western blot, *P. penneri* 60 recognized only fast migrating bands of *P. penneri* 63 LPS (Fig. 2d), which explains the weak reaction observed for this system in ELISA (Table 1). After the adsorption of *P. penneri* 60 antiserum by *P. penneri* 63 LPS, the titer of its reactivity with homologous LPS was slightly lower compared to the titer of non-adsorbed serum (Fig. 3a). In order to find out which part of LPS was recognized by the antibodies that remained in the adsorbed serum, the serum was checked with both tested LPSs in Western blotting (Fig. 3a). As expected, the adsorption procedure removed the anti-core-specific antibodies from the serum, which indicates that *P. penneri* 60 and 63 LPSs present one serotype of the core regions.

### *P. penneri* 103 antiserum

*P. penneri* 103 antiserum cross-reacted in ELISA only with one LPS and *P. penneri* 75 to the titer equal to that of homologous LPS (Table 1). In Western blot, similar strong reactions were observed for the serum with both high- and low-molecular-mass LPS species of the homologous strain and of the cross-reacting *P. penneri* 75 strain (Fig. 2e). After the adsorption of *P. penneri* 103 antiserum by *P. penneri* 75 LPS, only a small fraction of anti-LPS antibodies, reacting to the titer 1:400 in PIH and recognizing slow-migrating bands of *P. penneri* 103 LPS in Western blotting, remained in the serum (Fig. 3b).

### *P. penneri* 124 antiserum

**Fig. 4:**
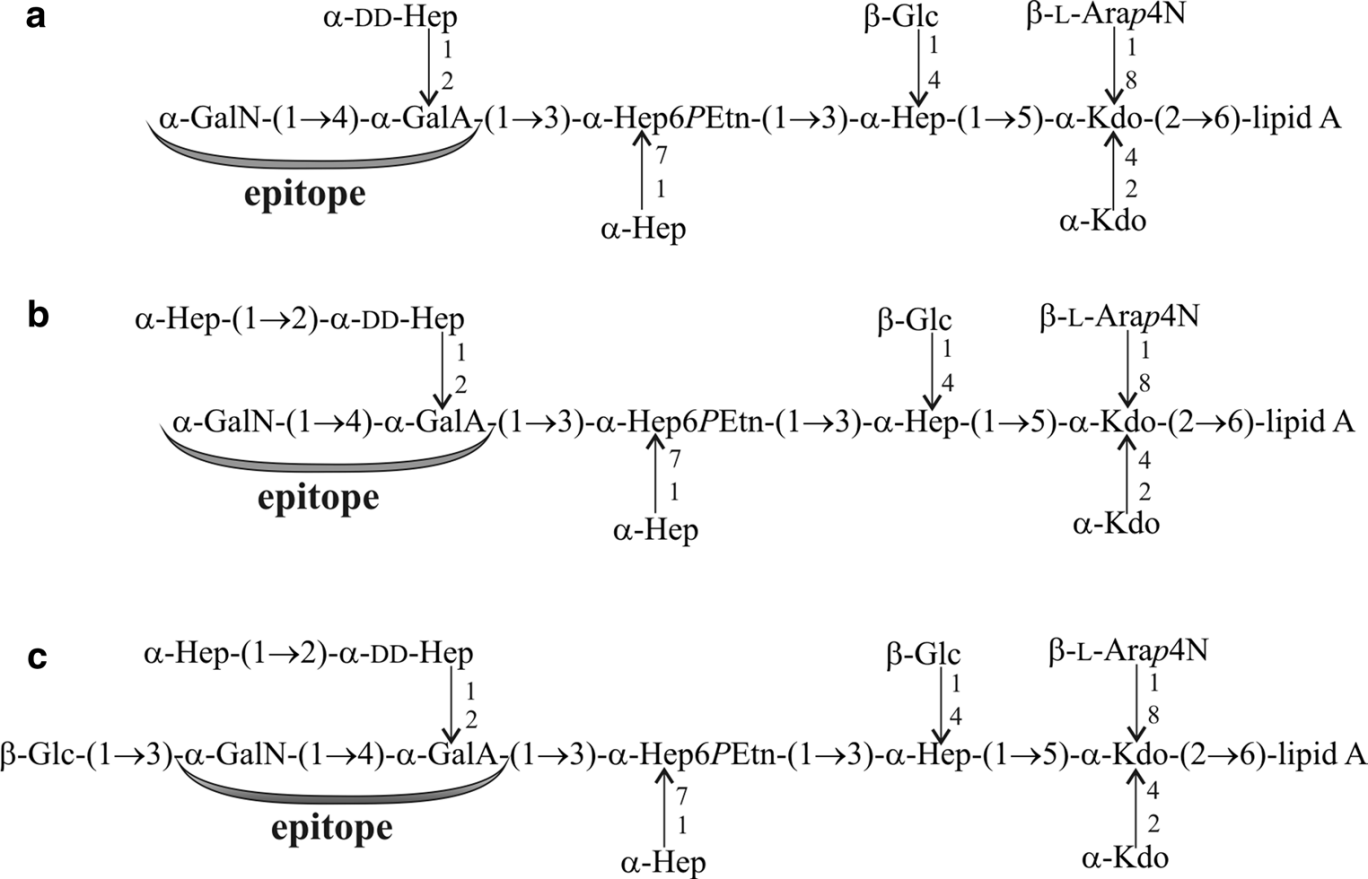
LPS core region structures of: **a** *P. penneri* 12, **b** *P. penneri* 13 and **c** *P. penneri* 26 LPSs [11, 16]; Ara*p*4N, 4-amino-4-deoxy-l-arabinopyranose; GalA, galacturonic acid; GalN, galactosamine; Glc, glucose; dd-Hep, d-*glycero*-d-*manno*-heptose; Kdo, 3-deoxy-d-*manno*-oct-2-ulosonic acid; and *P*Etn, 2-aminoethyl phosphate

*P. penneri* 124 antiserum is specific to the clinical rough strain, i.e., it contains the core-specific antibodies only. It was selected for the studies to check whether *P. penneri* 124 LPS presents the same core region serotype as the type strain of the species, *P. penneri* 12 (American Type Culture Collection 33519) with the known core region structure (Fig. 4) [11]. The previous serological studies conducted with the use of the *P. penneri* 13 antiserum showed its strong cross-reactivity with *P. penneri* 12, 124, 26 and 112 LPSs, reacting to the same reactivity titer (1:16.000) as *P. penneri* 13 LPS [16]. To confirm the previously obtained results, the *P. penneri* 13, 26 and 112 LPSs were additionally selected to be included in the present work. As was expected, the *P. penneri* 124 antiserum reacted in ELISA identically with *P. penneri* 12, 124, 13 and 112 LPSs, but the reaction with *P. penneri* 26 LPS was characterized by the lowest intensity (Table 1). In Western blot, the *P. penneri* 124 antiserum recognized the fast migrating bands of all tested LPSs (Fig. 2f). In contrast to ELISA results, *P. penneri* 112 LPS reacted more weakly than homologous LPS. The adsorption procedure of the tested serum completely abolished all reactions previously observed for non-adsorbed serum (data not showed).

## Discussion

The serological results of the present study have allowed classifying 22 LPSs of *P. penneri* 2, 11, 12, 16, 17, 18, 19, 24, 28, 31, 35, 36, 38, 60, 63, 75, 100, 103, 107, 114, 115 and 124 into one of the five new core serotypes (Table 2, below the middle line). Four of the antigen groups contain LPSs with determined core region structures [11] (in Table 2 marked with*), and one contains only LPSs (*P. penneri* 60 and 63) with structurally unknown core regions. Table 2 includes all serotypes of LPS core regions formed on the basis of the results (Table 1; Figs. 2, 3) and previous serological studies [16, 17, 19, 20]. The studies were possible to perform owing to a unique set of anti-core sera obtained by: (1) the adsorption of serum against the bacterial whole cells by the LPS possessing the same O-polysaccharide as the strain homologous to the serum and different core region serotypes, (2) the immunization of rabbit with the conjugate of core oligosaccharide with diphtheria toxoid and (3) the immunization of rabbit with the whole cells of rough strains or of smooth strains but having the majority of LPS molecules unsubstituted with O-polysaccharide chains.

**Table 2:** First typing scheme based on the core region serological specificity of 35 *P. penneri* and three *P. mirabilis* LPSs

No.	The core serotype presented by the LPSs	Other representatives of core region serotypes
1.	*P. penneri* 7* (O61)	*P. penneri* 14* (O59) and 15* (O52)
2.	*P. penneri* 8* (O67)	*P. penneri* 34 (O65) and 133 (O61)
3.	*P. penneri* 42* (O71)	*P. mirabilis* 51/57* (O28) and R14/S1959
4.	*P. penneri* 13* (rough form)	*P. penneri* 112 (O8)
4a.	Subgroup	*P. penneri* 26* (O31a)
5.	*P. penneri* 37* (rough form)	*P. penneri* 44* (rough form)
6.	*P. mirabilis* R110* (rough mutant)	*P. penneri* 47 (O59)
7.	*P. penneri* 28 (O31a,b)	*P. penneri* 16*, 18* (O17) and 31 (O19a,b)
8.	*P. penneri* 17* (O8)	*P. penneri* 2* (O66), 11* (O58), 19*, 24, 35, 36, 38, 100, 114 (O64a,b,c), 107* (O8) and 115 (O58)
9.	*P. penneri* 60 (O70)	*P. penneri* 63 (O68)
10.	*P. penneri* 103* (O73a,b)	*P. penneri* 75 (O73a,c)
11.	*P. penneri* 124 (rough form)	*P. penneri* 12* (O58)

LPS of the structurally defined core regions

The first representatives of each core region serotype are homologous to the respective antiserum used in the studies

() O serotype of LPS

Assigning the *P. penneri* LPSs to the appropriate core serotype has not always been easy to perform. In some cases, one LPS cross-reacted with the appropriate serum similarly to homologous LPS in one method and weaker in another technique, e.g., *P. penneri* 112 LPS reacted with *P. penneri* 124 antiserum to the same titer as homologous LPS (Table 1) and in Western blot more weakly than homologous LPS (Fig. 2f). The differences in the antisera reactivity titers within one group of LPSs may also result from the different number of LPS molecules substituted and unsubstituted with OPS-chains, thus from the different access of antibodies to the core region.

Although *P. penneri* 28 LPS possesses the core region with an unknown structure, it was selected as a representative of serotype no. 7 (Table 2) because it was possible to obtain the serum specific to its core region. The reactions observed for this serum in Western blot (Fig. 2a) with *P. penneri* 28, 16, 18 and 31 LPSs were the most diverse in their intensity. The LPS exhibiting the most distinguished reaction was *P. penneri* 18 LPS, for which the ladder-like banding pattern was observed at the level corresponding to the higher-molecular LPS species. Smearing which appeared for *P. penneri* 28 and 16 LPSs at the level, typical for higher-molecular mass species, concerned unspecific reactions since both LPSs have no common fragments in the OPS parts [9]. What is more, *P. vulgaris* 55/57 OPS which is known to be structurally identical to the O-antigen of the *P. penneri* 28 LPS has not reacted with the tested serum (Fig. 2a). The method, which occurred to be very useful for proving that all tested antigens present a common core region serotype, was the serum adsorption. It allowed abolishing the reactions observed previously with the unadsorbed serum (Table 1; Fig. 2a). The procedure of antiserum adsorption, by LPS molecules coated on SRBC as well as on the bacterial cells, is a proven method for assessing serological similarities between antigens. Even though slight differences in reactivities have been observed for a group of LPSs with unadsorbed serum, the adsorption procedure usually appears to be helpful in explaining disputable results [16, 17].

The serum adsorption procedure also enabled classifying 11 LPS to the core serotype presented by *P. penneri* 17 LPS. It is the most numerous group among the analyzed LPSs, having one serotype of the core region, including five LPSs of the determined core region structures (*P. penneri* 2, 11, 17, 19 and 107). The structural studies showed that the core regions of the mentioned LPSs possess a unique type of linkage [(*1S*)-Gal*o*NAc-(1 → 4,6)-α-GalN] occurring also in OSs of a few *Proteus* spp. LPSs, which had not previously been reported in natural glycopolymers [11]. This linkage probably occurs in OSs of the remaining *P. penneri* LPS presenting the same serotype as the *P. penneri* 17 core region.

LPSs *P. penneri* 60 and 63 present a core region serotype new for *P. penneri* strains tested so far [16, 17, 19, 20]. *P. penneri* 60 antiserum cross-reacted only with the low-molecular-mass species of *P. penneri* 63 (Fig. 2d). This result also stays in agreement with the structural data showing that in OPSs of both LPSs there is no common fragment except for the *N*-acetyl-fucosamine residue [-3)-α-l-Fuc*p*NAc-(1-] [9, 22]. It should be remembered that, in contrast to the anti-core sera, O-specific immunoglobulins predominate in the sera specific to the whole bacterial cells, including *P. penneri* 60 antiserum. It was reflected in ELISA by the higher titer of the serum with homologous LPS than that observed for *P. penneri* 63 LPS (Table 1). *P. penneri* 63 LPS was assigned to the core region serotype presented by *P. penneri* 60 LPS on the grounds of the Western blot results after the serum adsorption by *P. penneri* 63 LPS molecules (Fig. 3a). Abolishing the bands corresponding to the reactions with low-molecular-mass species of both tested LPSs and leaving the ladder-like banding pattern typical for the serum reaction with high-molecular-mass species of *P. penneri* 60 LPS (Fig. 3a, in a frame) clearly confirmed the suggestion that these LPSs present a similar serotype of the core region.

*P. penneri* 103 LPS was found to present the same core region serotype as *P. penneri* 75 LPS on the basis of the Western blot results obtained after the adsorption of *P. penneri* 103 antiserum by *P. penneri* 75 LPS (Fig. 3b). Only a slight reaction with slow-migrating bands of *P. penneri* 103 LPS was observed. Comparing the OPSs structures of both LPSs, it can be noticed that they differ only in the lateral substituents of the glucose residue and probably the lateral group (Etn*P* in *P. penneri* 103 OPS) was recognized by the immunoglobulins remaining in anti-*P. penneri* 103 serum after its adsorption by *P. penneri* 75 LPS (d-Glc residue except Etn*P*) (Fig. 3b, the reaction in a frame) [23].

The *P. penneri* 124 strain possesses LPS with a structurally unidentified core region, but its rough form decided about its selection for the studies. All serological data, the previous [16] and present ones (Table 1; Fig. 2f), indicate the *P. penneri* 124 LPS core region as serologically identical to the OS of *P. penneri* 12 LPS with a known core region structure (Fig. 4). Analyzing the results of *P. penneri* 124 antiserum and the previous results obtained for *P. penneri* 13 antiserum [16] in relation to the core region structures of *P. penneri* 12, 13 and 26 LPSs (Fig. 4) [11], it was confirmed that the common fragment of the core regions: α-GalN-(1 → 4)-α-GalA may be responsible for the observed cross-reactions. The results obtained for *P. penneri* 124 antiserum, after its adsorption by the tested *P. penneri* LPSs (data not showed), differ insignificantly from those obtained for *P. penneri* 13 antiserum [16]. Adsorption of *P. penneri* 13 antiserum with *P. penneri* 12 and 124 LPSs left a small fraction of antibodies (reciprocal titer 1.600) reacting with *P. penneri* 13, 112 and 26 LPSs, which was not observed in the present studies (all antibodies against *P. penneri* 12 and 124 LPSs were removed). It had been shown previously that those antibodies probably recognized the α-Hep-(1 → 2)-α-dd-Hep fragment, which is present in the core region of *P. penneri* 13 (probably of 112) and 26 LPSs but absent from *P. penneri* 12 LPS (probably from *P. penneri* 124) (Fig. 4). The weaker reaction of *P. penneri* 124 antiserum with *P. penneri* 26 LPS (Table 1; Fig. 2f) can be explained by: (1) the lack in the serum of the antibodies binding to the heptose disaccharide, (2) the additional terminal Glc residue (present only in the OS of *P. penneri* 26 LPS), absent from the outer core region of the remaining LPSs tested (Fig. 4), which may hamper binding the antibodies to the α-GalN-(1 → 4)-α-GalA fragment. However, this small structural difference in the *P. penneri* 13 and 26 core regions had not been shown to result in decreasing the titer of *P. penneri* 13 antiserum reactivity with *P. penneri* 26 LPS, compared to the titer in the homologous system [16]. Thus, the *P. penneri* 26 LPS was classified into the group of LPSs (with one core region serotype) represented by *P. penneri* 13 LPS as its subgroup 4a (Table 2).

Serological classification of *Proteus* spp. strains is not a typing method commonly used in clinical laboratories. However, the serological classification scheme based on the *P. penneri* core region provides useful information necessary for assigning the clinical isolates, not only from *P*. *penneri* species, to an appropriate core region serotype. Further extension of the scheme with other representatives may form a database, which, together with the scheme of *Proteus* LPS O-types, will show which core region serotype dominates within an area where the tested isolates come from. This may help in the selection of antigens presenting the most common O and R serotypes and serve as a tool to identify common epitopes among strains which may upon immunisation lead to the formation of cross-reactive and cross-protective antibodies against the core region as shown in one example for *E. coli* [24].

## Abbreviations

ELISA: Enzyme-linked immunosorbent assay
LPS: Lipopolysaccharide
OPS: O-specific polysaccharide
OS: Oligosaccharide
PBS: Phosphate-buffered saline
PIH: Passive immunohemolysis
SDS-PAGE: Sodium dodecyl sulfate-polyacrylamide gel electrophoresis
SRBCs: Sheep red blood cells
